# Emotional and Social Impact of Halitosis on Adolescents and Young Adults: A Systematic Review

**DOI:** 10.3390/medicina59030564

**Published:** 2023-03-14

**Authors:** Raluca Briceag, Aureliana Caraiane, Gheorghe Raftu, Razvan Mihai Horhat, Iulia Bogdan, Roxana Manuela Fericean, Luai Shaaban, Malina Popa, Bogdan Andrei Bumbu, Melania Lavinia Bratu, Marius Pricop, Serban Talpos

**Affiliations:** 1 Faculty of Dental Medicine, Ovidius University of Constanta, 7 Ilarie Voronca Street, 900684 Constanta, Romania; 2 Department of Oral Rehabilitation, Faculty of Dental Medicine, Ovidius University of Constanta, 900684 Constanta, Romania; 3 Department of Conservative Dentistry and Endodontics, Faculty of Dental Medicine, “Victor Babes” University of Medicine and Pharmacy Timisoara, Eftimie Murgu Square 2, 300041 Timisoara, Romania; 4 Department XIII, Discipline of Infectious Diseases, University of Medicine and Pharmacy “Victor Babes” Timisoara, Eftimie Murgu Square 2, 300041 Timisoara, Romania; 5 Doctoral School, “Victor Babes” University of Medicine and Pharmacy Timisoara, Eftimie Murgu Square 2, 300041 Timisoara, Romania; 6 Faculty of General Medicine, Baskent University, Fatih Sultan, Ankara 06790, Turkey; 7 Department of Pedodontics, Faculty of Dental Medicine, “Victor Babes” University of Medicine and Pharmacy Timisoara, Revolutiei Boulevard 9, 300041 Timisoara, Romania; 8 Department of Dental Medicine, Faculty of Medicine and Pharmacy, University of Oradea, 410073 Oradea, Romania; 9 Department of Psychology, Faculty of General Medicine, “Victor Babes” University of Medicine and Pharmacy Timisoara, Revolutiei Boulevard 9, 300041 Timisoara, Romania; 10 Discipline of Oral and Maxillo-Facial Surgery, Faculty of Dental Medicine, “Victor Babes” University of Medicine and Pharmacy Timisoara, Revolutiei Boulevard 9, 300041 Timisoara, Romania

**Keywords:** halitosis, oral health, quality of life, adolescent, young adult, socioeconomic factors

## Abstract

*Background and Objectives*: Halitosis is a condition characterized by unpleasant breath smell that is starting to receive serious scientific attention, considering it reflects on people’s social and personal life. While most studies focus on the prevalence of halitosis, its medical etiology, and the psychological impact on adults, there is a lack of evidence regarding the social impact of halitosis on the younger population. Therefore, this systematic review aimed to observe the social and emotional impact that halitosis has on adolescents and young adults. *Materials and Methods*: The review followed the PRISMA protocol, and four electronic databases (Scopus, Scholar, Web of Science, and ProQuest) were searched. From a total of 593 studies retrieved, only 6 were included in the study after assessing the eligibility criteria. *Results*: The main results showed that the levels of self-reported halitosis ranged from 23.1% to 77.5%, with an average of 44.7%, indicating a significant heterogeneity among the studies reporting this issue. Adolescents and young adults who experienced bad breath were feeling more anxious and depressed according to the non-standardized questionnaires and the standardized questionnaires (OHIP-14 and SCL-9-R). The respondents were isolated from social interactions and consequently had lower self-esteem and impaired quality of life. *Conclusions*: The conclusions drawn indicate the need for action on a medical level, as well as on a psychological level, in order to improve people’s oral health and help them navigate through the difficulties of maintaining social interactions as they live with halitosis.

## 1. Introduction

Halitosis, also known as oral malodor or bad breath, has a worldwide prevalence ranging from 22% to 50% [1], representing the third most common reason for contacting a dentist [2]. Although there are a series of factors that can contribute to the presence of halitosis, such as otorhinolaryngological diseases [2], gastrointestinal disorders [3], metabolic disorders, or chronic medication [4], in most cases, halitosis has an oral etiology with incorrect tongue brushing being the main factor that leads to this condition, followed by other intra-oral factors such as unstimulated salivation volume, periodontal conditions, caries, fixed orthodontic brackets, or debris accumulation [4,5,6,7].

The current literature classifies this condition into three categories: genuine halitosis, pseudo-halitosis, and halitophobia [7,8]. Genuine halitosis indicates the presence of malodor as a result of dental investigations such as organoleptic tests with sulfur portable reading devices [7]. On the other hand, pseudo-halitosis and halitophobia cannot be measured objectively, being described only by a subjective complaint by the person who seeks medical help [2,9]. While genuine halitosis requires a set of measures to control it, pseudo-halitosis can be improved through simple oral hygiene measures [8]. Halitophobia, on the other hand, implies the psychological dimension more, as people that believe they are suffering from halitosis tend to feel this way even after they take measures to improve their oral health [8].

Regardless of its type, halitosis has a great impact on a person’s quality of life, making it difficult to navigate through social interactions, as it is a condition that is highly noticed by other individuals and close contacts, and less by the person that experiences it [2,10]. Following the social difficulties that arise from having this condition, people may experience a series of mental health problems, such as depressive or anxious symptoms, reduced self-esteem, and social isolation [6,11,12]. Being a condition that can be directly perceived by other people, it often leads to social discrimination, stigma [13,14], and affects perceived body image and self-confidence [15]. In other words, the physiological impact is less significant compared to the psychological barriers that are created. The affected individuals with halitosis not only experience difficulties when it comes to their everyday lives and people with whom they come in contact with, but also when it comes to their intrapersonal life, as there can be a significant decline in their mental health [6].

Although there is a growing number of systematic reviews and meta-analyses that focus on the prevalence of halitosis, its medical factors, and potential treatment [1,16,17,18,19], only a few of them target the psychosocial aspects of this condition [10,12], although many studies state the major effect that it has on a person’s quality of life [6,9,11]. When it comes to younger populations, there are no systematic reviews that describe the psychosocial impact that halitosis has on this target group, although this segment of age can be very vulnerable when confronted with social interactions in their private life or in school settings [2]. Thus, the present review focuses on the emotional and social impact of halitosis on adolescents and young adults.

## 2. Materials and Methods

### 2.1. Study Design and Search Strategy

This systematic review was conducted according to the list of Preferred Reporting Items for Systematic Reviews (PRISMA) guidelines [20] registered in the Open Science Framework (OSF) platform. For this systematic review, two study participants (I.B. and R.M.F.) searched for all studies published up to and until November 2022 in four electronic databases (Scholar, Scopus, Web of Science, and ProQuest Central). All articles were screened independently by the two reviewers following the eligibility criteria. In case of disagreement, a third reviewer (R.M.H.) assessed the articles to reach a conclusion The search terms used in the databases were: “Halitosis”, “Humans”, “Social rejection”, “Quality of life”, “Oral health”, “Mouth diseases”, “Socioeconomic factors”, “Social behavior”, “Social impact”, and “Social interaction”; the Boolean operators AND-OR were used to refine the search. To narrow the search results and gather relevant articles on this topic, in some electronic databases, namely Web of Science and ProQuest Central, the term halitosis was restricted to abstracts. Reference lists from the retrieved articles were manually examined for relevant information. There were no search restrictions on the year of publication and language, although all the extracted articles were written in English.

### 2.2. Study Selection and Data Extraction

In terms of eligibility criteria, articles included in the systematic review had to match the following inclusion criteria: (1) report the presence of halitosis either through clinical examinations such as organoleptic tests or through self-reported questionnaires; (2) evaluate the impact that it has on the social or emotional aspects of one’s life using validated scales (standardized tests) or specific questions (unstandardized surveys); (3) studies should be performed on a cohort comprising adolescents and young adults ranging from 10 to 26 years of age; (4) participants must be without a medical diagnosis known as etiology for halitosis; and (5) the inclusion criteria was limited to observational studies that comprised cross-sectional, case-control, and cohort studies. The exclusion criteria for the literature search comprised (1) book chapters, reviews, editorials, or case reports; (2) studies including patients older than 26 years old; and (3) studies that did not survey the social impact of halitosis. The variables considered for inclusion included the following: country of the study, number of participants, age, gender, frequency of halitosis, study design, study aims, halitosis assessment, impact assessment, and main study results. The study selection process involved removing duplicated records, abstract screening, and full-text screening considering all the eligibility criteria described.

### 2.3. Quality Assessment

Following the NHLBI-published Study Quality Assessment Tools, two researchers evaluated information from existing articles and reported results individually. The tools are unique to research designs and screen for any methodological or operational problems. The Quality Assessment Tool for Observational Cohort and Cross-Sectional Investigations was used for the remaining studies [21]. For each of the 14 questions for study evaluation, “Yes” replies were worth 1 point while “No” and “Other” responses were worth 0 points. The final quality score was then calculated. Therefore, investigations with a rating from 0 to 4 were deemed to be of low quality, research with a grade between 5 and 9 was deemed to be of acceptable quality, and investigations with a score of 10 or more were deemed to be of good quality.

## 3. Results

### 3.1. Study Characteristics

**Figure 1:**
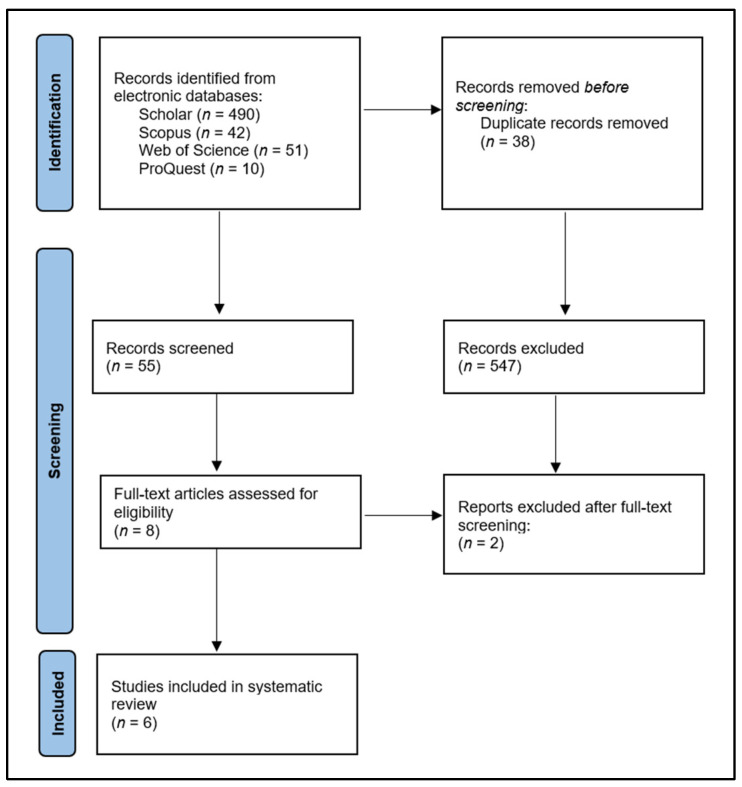
PRISMA Flow Diagram.

The initial search generated 593 records, of which 38 were duplicates. After screening the abstracts, eight articles were assessed in full-text, where two articles were excluded for not meeting the age criteria and the symptoms criteria, resulting in 6 articles [22,23,24,25,26,27] being included in the systematic review, as seen in Figure 1. Included studies took place in Brazil [22,26], India [25,27], Rwanda [23], and Nigeria [24]. The cohort sample size was relatively large, ranging from 200 participants [25] to 736 participants [22]. Age categories were distributed equally, with half of the studies focusing on adolescents [22,24,26] and the other half on young adults [23,25,27], along with gender, since both female and male patients were distributed almost equally in each study, apart from one that did not report gender proportions in the study groups [24].

The majority of the studies used self-reported questionnaires or specific questions to assess the presence of halitosis [22,23,24,26] and only two of them used organoleptic measures [25,27]. Questionnaires used to evaluate the presence of halitosis comprised items such as “Do you have bad breath?” [22], while the ones that used organoleptic tests implied medical devices that evaluate the presence of bad breath [25,27]. One study used FitScan Breath Checker, which is a palm-sized monitor that measures compounds responsible for breath odors [25], and another one used Breath Alert, a device that measures the breath odor of the person who blows into it [27].

**Figure 2:**
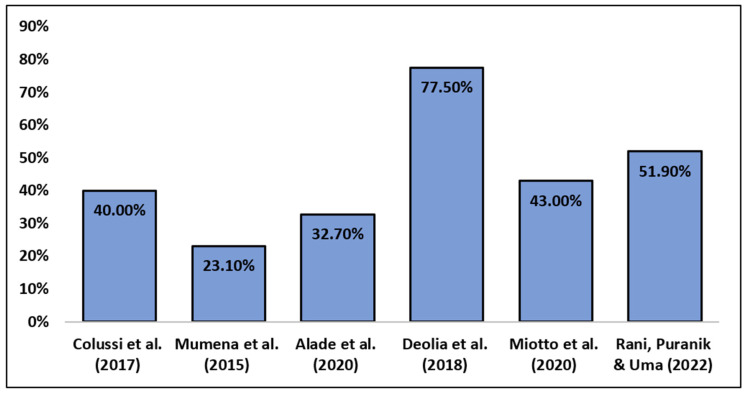
Frequency of self-reported halitosis [22,23,24,25,26,27].

In terms of social and emotional impact, three of the studies evaluated the overall quality of life [22,24,26], two of them evaluated the social and psychological consequences of bad breath [23,25], and one of them evaluated the psychological impact from a mental health disorder perspective [27]. Additionally, two studies did not use a psychometric scale and relied only on the specific question that regards psychosocial aspects of halitosis [23,25]. Studies that used a validated scale relied on the Oral Health-Related Quality of Life Scale (OHRQoL), which focuses on several dimensions such as functional limitations, physical pain, psychological discomfort, physical disability, psychological and social disability, and handicap [22,24,26]. The studies that did not opt for a validated scale used specific questions such as “Do you ever stay alone to avoid other people from feeling that you have bad breath?” [23], or “Bad breath affects your self-esteem or confidence?”, or statements such as “Relationships have failed because of your bad breath” [25]. One study used a psychodiagnostic scale, Symptom Checklist-90-Revised (SCL-90-R), that follows multiple pathological domains from neurotic psychopathologies such as anxiety and depression to psychotic ones [27]. All studies included had a cross-sectional design [22,23,24,25,26,27]. The demographic characteristics of the studies are found in Table 1. As most of the studies took into consideration other oral health problems aside from halitosis (i.e., malocclusion and decayed teeth), the number of people experiencing halitosis varied, as seen in Figure 2.

### 3.2. Impact of Halitosis

All studies included in this review found an important association between halitosis and quality of life, with an emphasis on the social dimensions. People who experienced halitosis reported having difficulties in their social life because of their bad breath, feeling more irritable and tense, and overall more self-conscious about their malodor [22,24,26]. In another study, both males and females felt that relationships had failed because of their bad breath, their academic goals were not met, and a lot of embarrassing situations arose from having this condition [25]. Furthermore, adolescents and young adults reported having lost friends because of halitosis, or they reported having been treated badly and thus implicitly started to avoid social interactions in order to protect themselves from the social discrimination and embarrassment from this type of situation [25].

When it comes to the estimated impact, one study reported that 48% of people suffering from halitosis had encountered social problems at school, 51.2% lost friends, and 60% avoided meeting people because they felt self-conscious about their breath [23]. Another study reported that 56.9% of people that have halitosis were hesitant in talking to people, 37.5% avoided meeting other people, 68.1% were avoided by their peers because of their bad breath, and overall, 46.3% felt their personal lives had been affected by malodor, and 64.4% felt their social lives had been affected by it [27]. Looking at quality of life, one study found that adolescents with self-reported halitosis were 1.48 times more likely to be more affected in their everyday life [22], while another study reported a 66.4% probability that adolescents with self-reported halitosis had a lower quality of life [24]. Miotto et al. reported that people who experience halitosis are 2.3 times more likely to experience a negative impact to their quality of life [26]. Apart from the social aspects, Rani et al. found that participants who had this condition had significantly poor psychological status, namely an increased score of depression [27]. When it comes to gender, a fraction of the studies did not report statistically significant differences between males and females [22,23], while others found that females were more affected by malodor [24,25,26,27]. Therefore, the impact of halitosis did not differentiate between the two targeted age groups. Table 2 shows the characteristics of the studies in terms of social and emotional impact.

## 4. Discussion

Halitosis is a condition experienced by many people, impacting their quality of life and restraining them from enjoying social interactions. Many studies highlight the need to address this issue, considering the potentially harmful effects that it may have on people’s quality of life [6,10,11]. In this systematic review, all the included studies found that halitosis negatively impacted adolescents and young adults on a social and psychological basis [22,23,24,25,26,27]. This is consistent with another systematic review and meta-analysis [10], which found halitosis to be associated with impaired quality of life related to oral health, although the studied population included older adults and the elderly. Other systematic reviews drew the same conclusions, stating that halitosis has a great impact on social aspects, leading people to withdraw from their social lives and leaving them with social anxiety, low self-esteem, depression, and broken relationships [1,18,28]. Another conclusion that has been stated by other reviews also emphasizes the fact that women tend to be more affected by halitosis [1,18], feeling more embarrassed and self-conscious and, thus, having a greater impact on their quality of life. These results put forward the need to address the psychological impact that this condition implies, given the fact that there are fewer studies that look at these aspects.

In terms of medical treatments, there are available options that have been found to be effective, such as photodynamic therapy [29] or active mouth rinse [30], as well as programs that are meant to improve the general public’s knowledge about oral health and effective ways to maintain it [30]. Apart from the general public, there are also clinical guidelines for dentists that can be used to treat halitosis effectively [31]. One study states that the first important thing in establishing a halitosis diagnosis is the identification of determining factors such as poor oral hygiene, periodontal diseases, or systemic illnesses [27]. Taking this into consideration, the considerable amount of information about bad breath and its treatments needs to be merged with guidelines that emphasize the social and emotional aspects so that people who suffer from it will not have a significant psychological decline that affects their daily lives. The issue has to be addressed in multiple contexts, from education about oral hygiene [4] to information about its associated factors such as tobacco or alcohol use [22], and lastly to symptoms that may arise because of the difficulties experienced, such as anxiety or depression [32].

Oral hygiene education plays a crucial role in preventing halitosis, or bad breath [33]. It is an essential aspect of oral health care that can help individuals maintain healthy gums and teeth, reduce the risk of oral diseases, and prevent bad breath. One of the main causes of halitosis is poor oral hygiene, which can result from the buildup of plaque, gum disease, and other oral health problems [34]. By educating individuals about the importance of proper oral hygiene practices, such as brushing and flossing regularly, using mouthwash, and visiting a dentist regularly, people can take steps to prevent these issues and reduce the risk of halitosis [35].

Moreover, oral hygiene education can also help to dispel common myths and misconceptions about oral health, such as the belief that bad breath is simply a natural part of aging or that it is not a serious health concern [36]. By providing accurate and evidence-based information, individuals can make informed decisions about their oral health and take the necessary steps to maintain good oral hygiene and prevent halitosis. In addition to individual prevention, oral hygiene education can also play a critical role in reducing the incidence of halitosis in communities and populations [37]. This can be achieved through community-based oral health programs, school-based oral health education, and other public health initiatives [38]. These programs can help to raise awareness of the importance of oral hygiene, promote healthy oral health habits, and prevent halitosis and other oral health problems. Additionally, oral hygiene education can also play a role in addressing disparities in oral health and halitosis, particularly in low-income or underprivileged communities where access to oral health care may be limited [39]. By providing education and resources in these communities, individuals can take control of their oral health and reduce their risk of halitosis and other oral health problems [40,41].

In this review, the studies included lacked proper instruments with good psychometric properties in evaluating social and emotional impact, as most of them relied on single questions with dichotomous answers [23,25] or used a simplified scale (OHIP-14) [22,24,26] that focused more on the overview of the quality of life and had a small number of questions that target the psychosocial dimensions; therefore, some nuances cannot be tracked. Another problem when using this instrument is that it was not constructed specifically for halitosis but for general oral health conditions, which include dental and oral disease [10]. The same thing was observed when evaluating the presence of halitosis: most studies relied on single questions for participants to self-report the condition, and only two of them used a proper medical instrument, an organoleptic test [25,27]. Furthermore, the studies were conducted in Brazil [22,26], India [25,27], Rwanda [23], and Nigeria [24], so there was no study conducted in the USA or Europe, which makes it difficult to extrapolate a conclusion for the general population, given the fact that cultures differ substantially in each region, so they may perceive and respond differently to bad breath.

Nevertheless, stomatologists and psychologists need to collaborate in order to educate their patients that suffer from bad breath, one by teaching the proper ways to maintain proper oral hygiene and the other by finding protective factors and by managing the psychological symptoms, from emotions to avoidant behaviors, in ways that will not destabilize a person’s mental health and coping mechanisms [32]. Although studies show that dental professionals are aware and educated about halitosis and its aspects, this knowledge needs to be addressed to the general public as well [4], considering the high prevalence of halitosis [1]. Future studies may concentrate on finding specific factors that contribute to the psychosocial impact of halitosis. For example, one study found neuroticism to be a risk factor while conscientiousness was a protective factor [32], so personality traits play a part in the way that malodor occurs and in the way that it is being managed. Another study describes how psychological problems may arise from poor oral behavior practices, as in depression; for example, the person may feel apathetic and implicitly may have difficulties with daily activities, including basic hygiene [27].

Another aspect that should be considered is interventions that are able to reduce the social stigma around this condition and bring forward adaptive coping strategies for people who are affected by halitosis. This could be done through educational programs, such as the ones used to spread general information about oral health and its relation to halitosis [30] or through specialized help, such as counseling or therapy [11]. Besides this, it is important to keep an eye on demographic characteristics, as studies note that, in many cases, these can interfere with results [1]. For example, low educational level and low income have been associated with a higher psychological impact of halitosis [33] along with gender, as it has been estimated that women tend to perceive a higher impact than men [1,18]. This demonstrates that halitosis has various risk factors that need to be rigorously researched in order to formulate a specific response to how this condition can be prevented and managed. The aspects that it implies need to be addressed by a multidisciplinary team in order to effectively prevent the condition and help people that suffer from it regain their autonomy in different aspects of their lives, including the social dimension [6].

The prevalence of halitosis, or bad breath, is a significant health concern in many countries, particularly in developing or underdeveloped nations [42]. The articles included in the analysis of halitosis may have overlooked the potential impact of the socioeconomic situation of these countries on the high incidence of the condition. This is a crucial issue that needs to be addressed and considered in the discussion sections of such articles. The socioeconomic situation of a country can have a profound effect on the health and wellbeing of its citizens [43]. In developing or underdeveloped nations, poverty, lack of access to clean water, and inadequate healthcare systems can lead to poor oral hygiene, which is one of the primary causes of halitosis. For example, people living in poverty-stricken areas may not have access to regular dental check-ups, or may not be able to afford proper oral hygiene products. This can lead to a buildup of plaque, gum disease, and other oral health problems that can cause bad breath [44].

Moreover, functional illiteracy and low levels of literacy can also play a role in the high prevalence of halitosis in developing countries. Many individuals in these countries may not have access to proper health education or may not be able to read or understand written instructions for oral hygiene practices. This can lead to poor oral hygiene and an increased risk of halitosis [45]. It is important for the authors of articles on halitosis to take these factors into consideration when analyzing the prevalence of the condition in developing or underdeveloped nations. Including discussion on the influence of the socioeconomic situation and literacy levels on halitosis can provide a more comprehensive understanding of the problem and help to inform prophylactic or treatment recommendations [46]. Additionally, it would be beneficial for authors to consider the cultural and societal attitudes towards oral hygiene in these countries. In some cultures, oral hygiene may not be given as much importance as it is in other countries [47]. This can also contribute to the high incidence of halitosis in developing or underdeveloped nations.

Considering the impact that socioeconomic status and literacy levels have on the prevalence of halitosis in developing and underdeveloped countries, there is a need for future medical research studies to investigate the root causes of this oral health problem. Research studies should explore the cultural practices, dietary habits, and access to oral hygiene resources that contribute to the development of halitosis in these countries. Furthermore, studies should evaluate the psychosocial aspects of halitosis and the impact it has on mental health, social connections, academic performance, and overall quality of life, particularly in younger populations with active social lives. To improve the prevention and treatment of halitosis, studies should identify the most effective behavioral changes, oral hygiene practices, and therapeutic interventions. By addressing these research gaps, studies should investigate protective factors that can improve mental health and social connections for individuals affected by halitosis.

The present systematic review has several limitations that should be taken into consideration when interpreting the results. Firstly, the small number of studies included may limit the generalizability of the findings to larger populations. While efforts were made to identify all relevant studies, it is possible that some data may have been missed, potentially leading to a biased selection of studies. Secondly, the heterogeneity in the reporting of results across studies may have influenced the accuracy and reliability of the findings. In particular, the inclusion of both subjective and objective assessments of halitosis may have introduced bias, as subjective assessments may be influenced by individual perceptions and biases. Third, the included studies reported different ways of evaluating the presence of halitosis, such as different measurement techniques and cut-off points for diagnosis. This heterogeneity may have affected the comparability of the studies and the generalizability of the findings. Furthermore, the quality of the included studies varied, with some studies presenting a higher risk of bias than others. As such, the findings of this systematic review should be interpreted with caution, and future studies should aim to address these limitations to provide more accurate and reliable evidence regarding the prevalence and impact of halitosis.

## 5. Conclusions

In conclusion, the socioeconomic situation and literacy levels in developing or un-derdeveloped countries can have a significant impact on the prevalence of halitosis. It was observed that halitosis is associated with isolation, low self-esteem, anxiety, and an overall feeling of embarrassment, which eventually leads to unstable mental health, poor social interaction, impediments in reaching academic goals, and an overall decreased quality of life, especially in the younger population with an active social life. As this condition may lead to an impairment in a person’s life, people need to become more aware of how halitosis can occur and what are the most efficient ways to prevent it and treat it. Therefore, future studies should evaluate the multiple psychosocial impacts of halitosis and raise awareness for this particular young population that is affected the most.

## Figures and Tables

**Table 1:** Demographic characteristics of studies.

Publication	Country	Quality Assessment	Number of Participants	Age	Gender	Frequency of Halitosis
Colussi et al. (2017) [22]	Brazil	Good	736	15–19 years old	56.1% females	40.0%
Mumena et al. (2015) [23]	Rwanda	Fair	354	23.5 mean age	48.6% females	23.1%
Alade et al. (2020) [24]	Nigeria	Good	361	14.1 mean age	59.6% females	32.7%
Deolia et al. (2018) [25]	India	Excellent	200	18–25 years old	N/A	77.5%
Miotto et al. (2020) [26]	Brazil	Fair	680	15–19 years old	59.8% females	43.0%
Rani, Puranil & Uma (2022) [27]	India	Good	320	21.5 mean age	40.6% females	51.9%

**Table 2:** Study characteristics.

Publication	Design	Study Aims	Halitosis Assessment	Impact Assessment	Results
Colussi et al. (2017) [22]	Cross-sectional	Impact of oral health on the quality of life	Self-reported halitosis using a single question answered with a Likert scale.	Oral Health Impact Profile (OHIP-14)	Self-reported halitosis impacts quality of life
Mumena et al. (2015) [23]	Cross-sectional	Prevalence of self-perceived halitosis, its effects to social life, and associated factors	Self-reported halitosis using a structured self-administered questionnaire	Specific questions related to the consequences of bad breath	Self-reported halitosis impacts social life
Alade et al. (2020) [24]	Cross-sectional	Prevalence and impact of self-reported halitosis on the oral health-related quality of life	Self-reported halitosis using a single question with a dichotomous answer	Oral Health Impact Profile (OHIP-14)	Self-reported halitosis impacts quality of life
Deolia et al. (2018) [25]	Cross-sectional	Psychosocial effects of halitosis	Organoleptic test—FitScan Breath Checker	Self-administered questionnaire which includes questions related to the psychological and social impact of halitosis	Halitosis impacts social and academic life
Miotto et al. (2020) [26]	Cross-sectional	The impact produced by oral disorders on the quality of life	Self-reported halitosis using a single question with dichotomous answer	Oral Health Impact Profile (OHIP-14)	Self-reported halitosis impacts quality of life (psychological and social dimensions)
Rani, Puranil & Uma (2022) [27]	Cross-sectional	Assessment of psychological status and self-perception of halitosis	Self-reported questions in halitosis, Organoleptic test—Breath Alert	Symptom Checklist-90-Revised (SCL-90-R)	Self-perceived halitosis impacts psychological dimensions (depressive symptoms)

## Data Availability

Not applicable.
